# Combined treatment of graft versus host disease using donor regulatory T cells and ruxolitinib

**DOI:** 10.1038/s41598-022-12407-x

**Published:** 2022-05-19

**Authors:** Alfonso Rodríguez-Gil, Virginia Escamilla-Gómez, Melanie Nufer, Félix Andújar-Sánchez, Teresa Lopes-Ramos, José Antonio Bejarano-García, Estefanía García-Guerrero, Cristina Calderón-Cabrera, Teresa Caballero-Velázquez, Clara Beatriz García-Calderón, Paola Hernández-Díaz, Juan Luis Reguera-Ortega, Nancy Rodríguez-Torres, Nuria Martínez-Cibrián, José Ignacio Rodríguez-Barbosa, Javier Villadiego, José Antonio Pérez-Simón

**Affiliations:** 1 Instituto de Biomedicina de Sevilla (IBiS/CSIC/Universidad de Sevilla), Seville, Spain; 2 Centro de Investigación Biomédica en Red en Cáncer-CIBERONC, Madrid, Spain; 3 Hospital Universitario Virgen del Rocío (HUVR), Seville, Spain; 4 Departamento de Fisiología Médica y Biofísica, Universidad de Sevilla, Seville, Spain; 5 Departamento de Medicina, Universidad de Sevilla, Seville, Spain; 6 Instituto de Biología Molecular, Área de Inmunología, Universidad de León, León, Spain; 7 Centro de Investigación Biomédica en Red Sobre Enfermedades Neurodegenerativas-CIBERNED, Madrid, Spain; 8 Instituto de Biomedicina de Sevilla (IBiS / CSIC) - CIBERONC and Departamento de Fisiología Médica y Biofísica, Universidad de Sevilla, Avda. Manuel Siurot s/n, 41013 Seville, Spain; 9 Present Address: Division of Blood and Marrow Transplantation, Stanford University, Stanford, CA USA

**Keywords:** Bone marrow transplantation, Haematological cancer

## Abstract

Donor derived regulatory T lymphocytes and the JAK1/2 kinase inhibitor ruxolitinib are currently being evaluated as therapeutic options in the treatment of chronic graft versus host disease (cGvHD). In this work, we aimed to determine if the combined use of both agents can exert a synergistic effect in the treatment of GvHD. For this purpose, we studied the effect of this combination both in vitro and in a GvHD mouse model. Our results show that ruxolitinib favors the ratio of thymic regulatory T cells to conventional T cells in culture, without affecting the suppressive capacity of these Treg. The combination of ruxolitinib with Treg showed a higher efficacy as compared to each single treatment alone in our GvHD mouse model in terms of GvHD incidence, severity and survival without hampering graft versus leukemia effect. This beneficial effect correlated with the detection in the bone marrow of recipient mice of the infused donor allogeneic Treg after the adoptive transfer.

## Introduction

The allogeneic transplantation of hematopoietic stem cells (allo-HSC) represents the best therapeutic option for many patients diagnosed with hematologic malignancies. Unfortunately, a significant proportion of patients receiving an allo-HSC develop acute or chronic Graft versus Host Disease (GvHD)^1–4^.

The JAK kinase inhibitor ruxolitinib has been used to ameliorate the effects of different inflammatory and myeloproliferative syndromes^5–13^, as the JAK kinases pathway plays a key role in the transmission of the cytokine signaling in inflammatory and immune processes. With this background, different studies evaluated the efficacy of ruxolitinib in the prophylaxis and treatment of acute or chronic GvHD first in preclinical models and subsequently in prospective randomized trials^14–25^. With the REACH trials, ruxolitinib has become the treatment of choice for steroid refractory acute and chronic GvHD^14,15,17^. Interestingly, the administration of ruxolitinib in mice developing GvHD increased regulatory T cells (Treg) as compared to the non-treated mice^16^.

The use of donor or third party derived Treg is another promising therapy against GvHD^26–36^. The Treg represent a subset of CD4+ T-cells with high expression of the IL2 receptor alpha (CD25) and are also characterized by a high expression of the transcriptional factor Forkhead box p3 (Foxp3)^37,38^. These cells are capable to modulate the immune responses produced by the effector immune cells, having a crucial role in the development of self-tolerance, and also in the induction of tolerance of the donor cells to the recipient tissues. The absence of Treg leads to severe autoimmune complications. The Treg can regulate both innate and acquired immune responses.

Based on the hypothesis that ruxolitinib favors the ratio of Treg to conventional T cells (Tcon) in previous studies, we propose that the combined use of donor derived Treg with ruxolitinib could help to achieve therapeutic effects with lower number of Treg, allowing also the tapering of immunosuppressive treatment in GvHD patients. To address this idea, we have conducted an in vitro study and a preclinical mouse model.

## Results

### In vitro effect of ruxolitinib on Treg

#### Ruxolitinib increases the natural Treg:Tcon ratio in vitro over time

**Figure 1:**
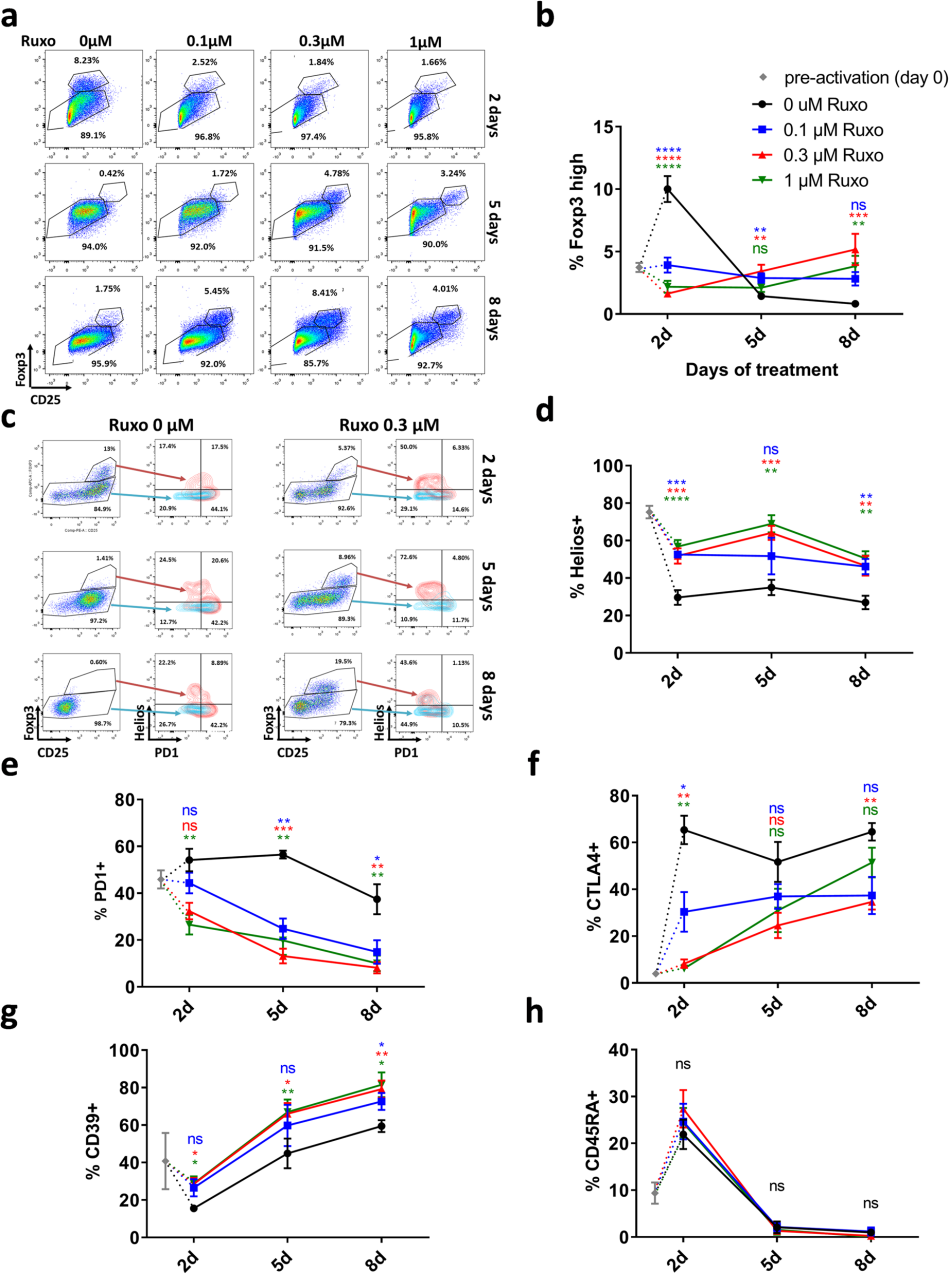
In vitro activated huPBMNCs in the presence of ruxolitinib show increased percentages of CD4+ Foxp3 high Helios+ cells along time. (**a**) Representative cytometry dot plots of huPBMNCs activated with anti-CD3 and anti-CD28 at different times and ruxolitinib doses. Dot plots show CD25 and Foxp3 staining of CD4+ gated cells. (**b**) Quantification of Foxp3^high^ cells of gated CD4+ huPBMNCs. Mean and standard error of the mean (S.E.M.) of a minimum of 4 independent experiments is represented for each condition. (**c**) Representative cytometry dot plots of huPBMNCs activated with anti-CD3 and anti-CD28 at different times and with 0 or 0.3 µM ruxolitinib. Dot plots show CD25 and Foxp3 staining of CD4+ gated cells, and density plots show Helios and PD1 staining. (**d**) Quantification of Helios+ cells of gated CD4+ Foxp3^high^ huPBMNCs. n = 6. (**e**) Quantification of PD1+ cells of gated CD4+ Foxp3^high^ huPBMNCs. n = 6. (**f**) Quantification of CTLA4+ cells of gated CD4 + Foxp3^high^ huPBMNCs. n = 4. (**g**) Quantification of CD39+ cells of gated CD4+ Foxp3^high^ huPBMNCs. n = 4. (**h**) Quantification of CD45RA+ cells of gated CD4+ Foxp3^high^ huPBMNCs. n = 4. One way ANOVA test with Dunnett’s correction for multiple comparisons p values are shown. Each treatment is compared with the 0 ruxolitinib control. p values: *< 0.05, **< 0.01, ***< 0.001, ****< 0.0001.

We tested the effect of the Jak1/2 inhibitor ruxolitinib in the proportion of regulatory T cells in in vitro cultures of human PBMNCs activated with anti-CD3 and anti-CD28 stimulation. As previously described^16^, increased concentrations of ruxolitinib reduced the activation of T cells (see decrease in CD25+ cells in Fig. 1a, Supplementary Fig. 1A). After 2 days of activation, CD4+ and CD8+ cells upregulated both CD25 and Foxp3 compared to non activated controls (Supplementary Fig. 1a). A population of CD4+ cells showed a higher expression than the CD8+ cells, and we identify them as Treg (Supplementary Fig. 1a). We detected a significant percentage of such CD4+ C25+ Foxp3^high^ Treg cells in non-treated cultures, while cultures treated with increasing amounts of ruxolitinib showed lower percentages (Fig. 1a,b, Supplementary Fig. 1A). However, at later time points (5 and 8 days), the percentage and absolute number of Foxp3^high^ in non-treated cultures dropped drastically, while in ruxolitinib treated cultures it increases over time. In our experiments, the optimal concentration of ruxolitinib to achieve a higher Treg:Tcon ratio was 0.3 µM (Fig. 1a,b, Supplementary Fig. 1B,C). We hypothesized that the Foxp3^high^ cells detected in the non-treated culture after two days were induced Treg (iTreg) generated after the hyperactivation of the culture, while the Foxp3^high^ detected in ruxolitinib treated cultures after longer periods where natural Treg (nTreg) with a stable phenotype. To test this hypothesis we stained the cultures with anti-Helios antibodies, as the Helios transcription factor is a marker of the thymic origin of the nTreg^39,40^. As shown in Fig. 1c,d, the ruxolitinib treated cultures were enriched in Helios+ Foxp3^high^ cells compared with non-treated controls, and this difference is maintained along the duration of the experiment. In freshly isolated PBMNCs, the percentage of Helios positive Treg cells is around 75% (Supplementary Fig. 2A).

We further characterized the Treg present in the culture. PD1 (Programmed cell Death 1) is implicated in the immune checkpoint, and in the case of Type 1 regulatory T cells (Tr1), is one of the mechanisms by which suppression is achieved^41^. On the other hand, it is also an exhaustion marker of activated effector T cells, and recently it has been described as a negative factor for Treg suppressive capacity in a lineage specific K.O. mouse model^42^. We found that PD1 expression is higher in Helios− Foxp3^high^ cells as compared to Helios+ Foxp3^high^ (Fig. 1c,e) showing an inverse correlation (Supplementary Fig. 2B). In freshly isolated, non-stimulated cells, most Foxp3+ cells are Helios+ and there is no correlation with PD1 expression (Supplementary Fig. 2A).

Cytotoxic T Cell Antigen 4 (CTLA4) is another immune checkpoint receptor that is required for Treg function, and for Tcon homeostasis^43^. While ruxolitinib reduced the expression of CTLA4 in early time points (Fig. 1f, Supplementary Fig. 2C), within higher doses, the expression was recovered over time.

CD39 is a marker that has been correlated to the suppression capacity of the Treg^44,45^, due to its catalytic activity producing extracellular adenosine. As shown in Fig. 1g, CD39 increases its expression along the culture in all the experimental conditions, but interestingly it was higher in ruxolitinib treated Treg as compared to non treated cells at all time points analyzed.

Finally, CD45RA expression is associated with a naïve phenotype, and in peripheral blood (PB) Treg it is used to identify a population which can be expanded in vitro maintaining the suppressive properties^46–48^. In our study, as shown in Fig. 1h, the CD45RA expression is lost in all populations studied along the culture, independently of the Helios expression. This indicates that all cells in the culture, including the thymic nTreg, are activated due to the anti-CD3 and CD28 stimulation, losing their naïve phenotype.

#### Ruxolitinib inhibits homing CCR9 and CCR5 and inflammatory CXCR3 receptors

**Figure 2:**
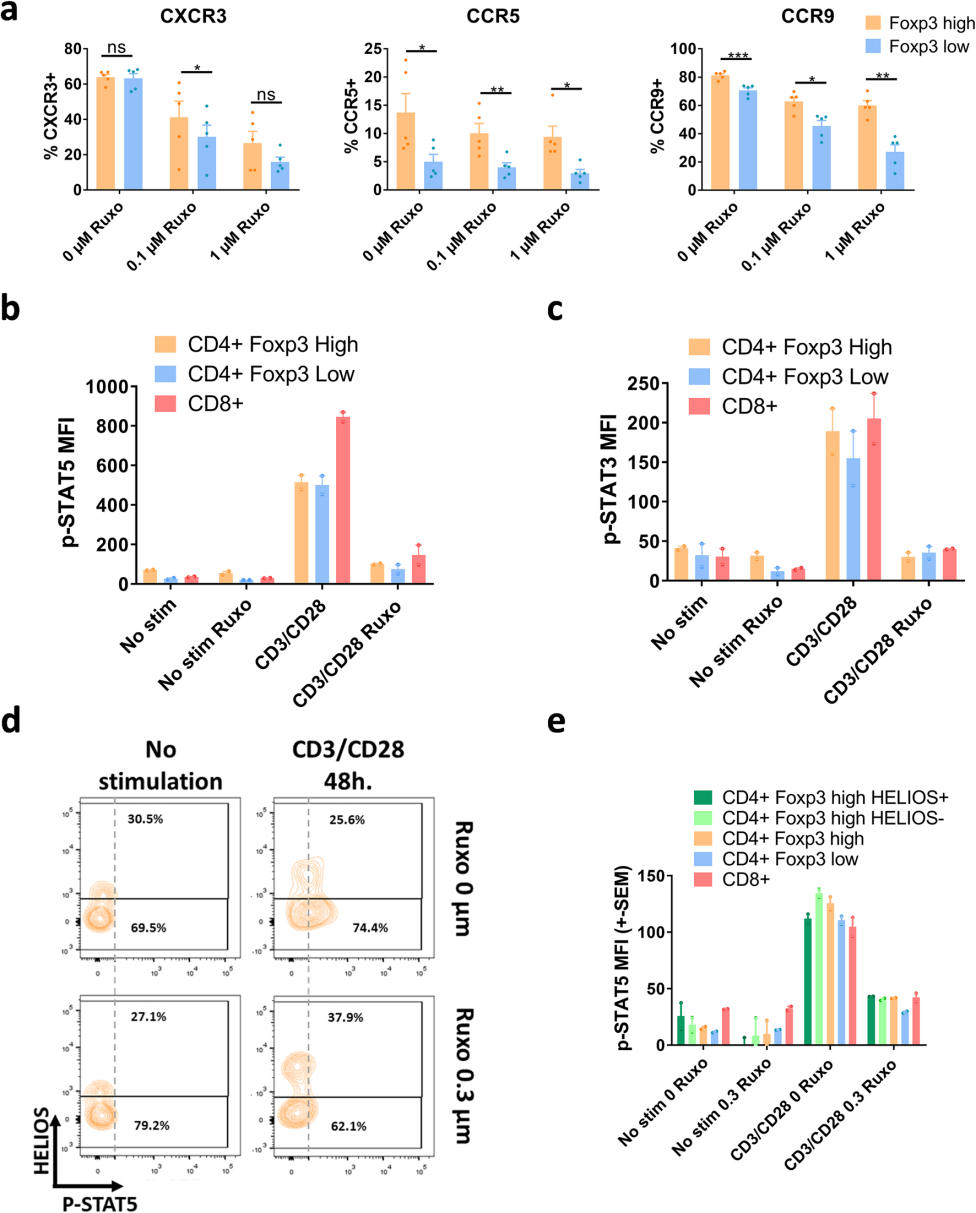
Effect of ruxolitinib on homing chemokine receptors and Stat phosphorylation. (**a**) Percentage of Cxcr3, Ccr5 and Ccr9 positive cells in CD4+ Foxp3^high^ and Foxp3^low^ gated cells after 48 h of anti-CD3/CD28 stimulation of huPBMNCs. Cells were treated with the indicated amounts of ruxolitinib from the beginning of stimulation. Mean and S.E.M. of 5 independent experiments is represented. (**b**) Quantification of intracellular Phospho-Stat5 cytometry. huPBMNCs were cultured for 48 h in the presence of 0 or 0.3 µM ruxolitinib, with or without anti-CD3 and CD28 stimulation. Cells were gated for CD4+ Foxp3^high^, CD4+ Foxp3^low^ and CD8+. Average and S.E.M. of the Median Fluorescence Intensity (M.F.I.) of two independent experiments with two technical replicates each are shown. (**c**) As in B, but Phospho-Stat3 staining was used instead. (**d**) Cytometry density plots of Phospho-Stat5 and Helios intracellular Staining of CD4+ Foxp3^high^ gated huPBMNCs cells, cultures as in B. (**e**) As in B, but in this case CD4+ Foxp3^high^ cells are also gated in Helios+ and Helios− cells. One representative of two independent experiments with two technical replicas each is shown. p values of a paired Student’s t-test are represented. p values: *< 0.05, **< 0.01, ***< 0.001, ****< 0.0001.

Another aspect which could affect the efficacy of the treatment with Treg is their capacity to migrate to the GvHD target organs. Ccr9, Ccr5 and Cxcr3 have been previously correlated with the migratory capacity of T cells to the gut under pathogenic conditions^49–51^. Of them, Cxcr3 has been shown to be downregulated by IFN-γ signaling disruption^52^, either by receptor elimination or by signal transduction inhibition with ruxolitinib. We determined the effect of ruxolitinib in these three chemokine receptors expression in CD4+ Foxp3^high^ cells compared to CD4+ Foxp3^low^ (Fig. 2a). Ruxolitinib reduces the expression of Cxcr3 and also of Ccr9 in Tcon and Treg, and Ccr5 only in Treg. However, in all cases, the expression of the three receptors was significantly higher in Foxp3^high^ than in Foxp3^low^ CD4+ T cells. This suggest that homing could be less affected by ruxolitinib in Treg as compared to Tcon.

#### Phosphorylation of Stat3 and Stat5 is decreased in both Treg and Tcon after ruxolitinib treatment

In previous studies, the effect of ruxolitinib on the phosphorylation of both Stat3^16^ and Stat5^53^ transcription factors in CD4+ T cells has been studied. In both cases, ruxolitinib reduces the phosphorylation of these signal transducers. On the other hand, it has been described the essential role of Stat5 for the development of Treg, while Stat3 is not required^54^. Interestingly, it has been proposed that Stat3 phosphorylation depends mainly on Jak1 and 2, while Stat5 is targeted by Jak2 and Jak3^55^. Thus, we decided to test whether ruxolitinib affected the phosphorylation of Stat5 and Stat3 in Treg and Tcon. After 48 h of anti-CD3 and anti-CD28 stimulation, a strong Stat5 Phosphorylation in CD8+, CD4+ Foxp3^low^ and CD4+ Foxp3^high^ was observed (Fig. 2b), which was completely abolished by ruxolitinib treatment in all cases. The same was true for Stat3 Phosphorylation (Fig. 2c). We checked whether or not there was any difference in Helios+ and Helios- cells, and the same result was observed for both populations (Fig. 2d,e).

#### Ruxolitinib does not hamper the suppressive capacity of regulatory T cells

**Figure 3:**
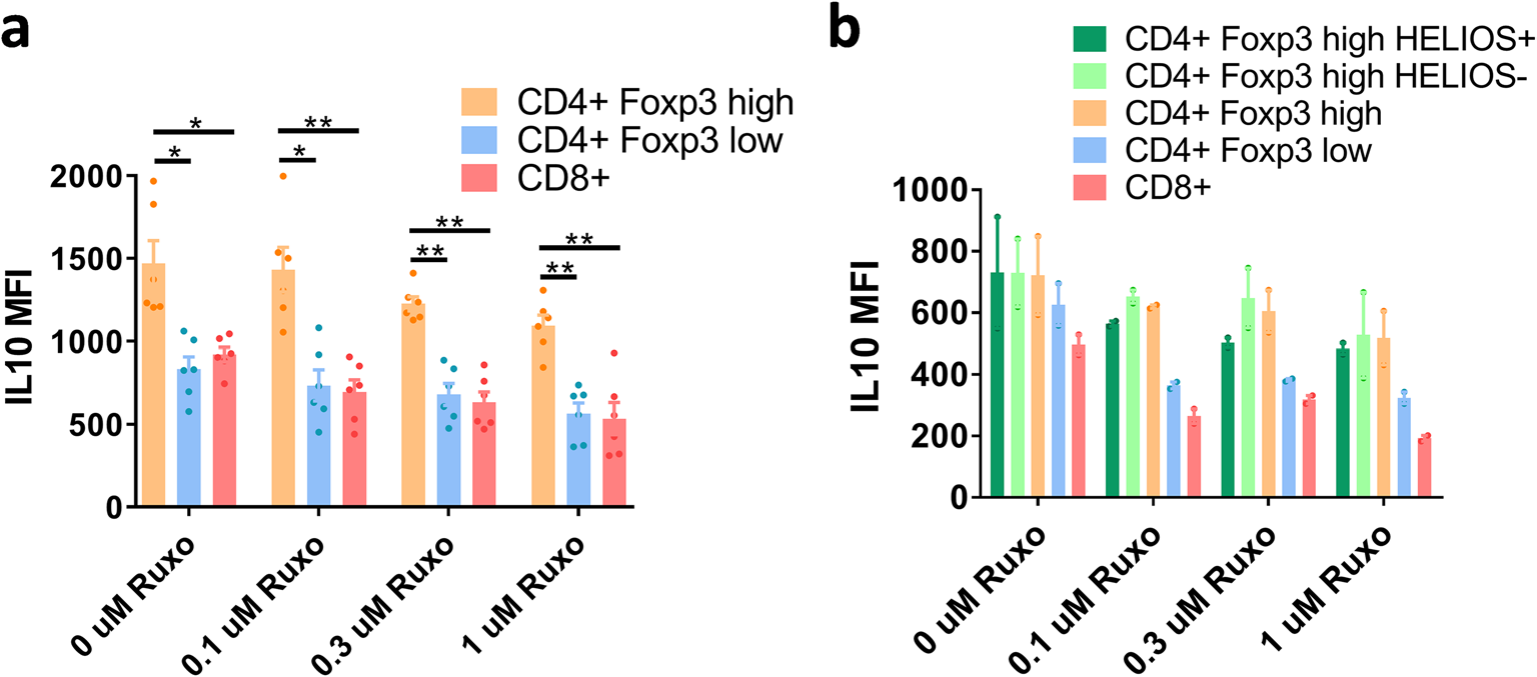
Quantification of intracellular IL10. (**a**) Quantification of intracellular IL10 staining of cells gated for CD4+ Foxp3^high^, CD4 + Foxp3^low^ and CD8+. huPBMNCs were cultured for 48 h in the presence of 0, 0.1, 0.3 or 1 µM ruxolitinib, with anti-CD3 and CD28 stimulation. Average and S.E.M. of the M.F.I. of five independent experiments are shown. p values of a one-way ANOVA test with Dunnett’s correction for multiple comparisons are shown. *< 0.05, **< 0.01, ***< 0.001, ****< 0.0001. (**b**) As in A, but CD4 + Foxp3^high^ cells are also gated for Helios+ and Helios− staining. A representative experiment of two biological replicates with two technical replicates is shown.

To functionally test the suppressive capacity of the ruxolitinib-treated Treg, we first tested IL-10 production in CD4+ Foxp3^high^ cells present in PMNCs cultures after 2 days of activation and treatment with different concentrations of ruxolitinib (Fig. 3a), compared with CD4+ Foxp3^low^ and CD8+ cells. CD4+ Foxp3^high^ showed a higher amount of IL-10 staining at all concentrations of ruxolitinib, with a decrease in IL-10 intracellular staining with increasing concentrations of ruxolitinib. This was true for both Helios+ and Helios− CD4 + Foxp3^high^ cells (Fig. 3b).

**Figure 4:**
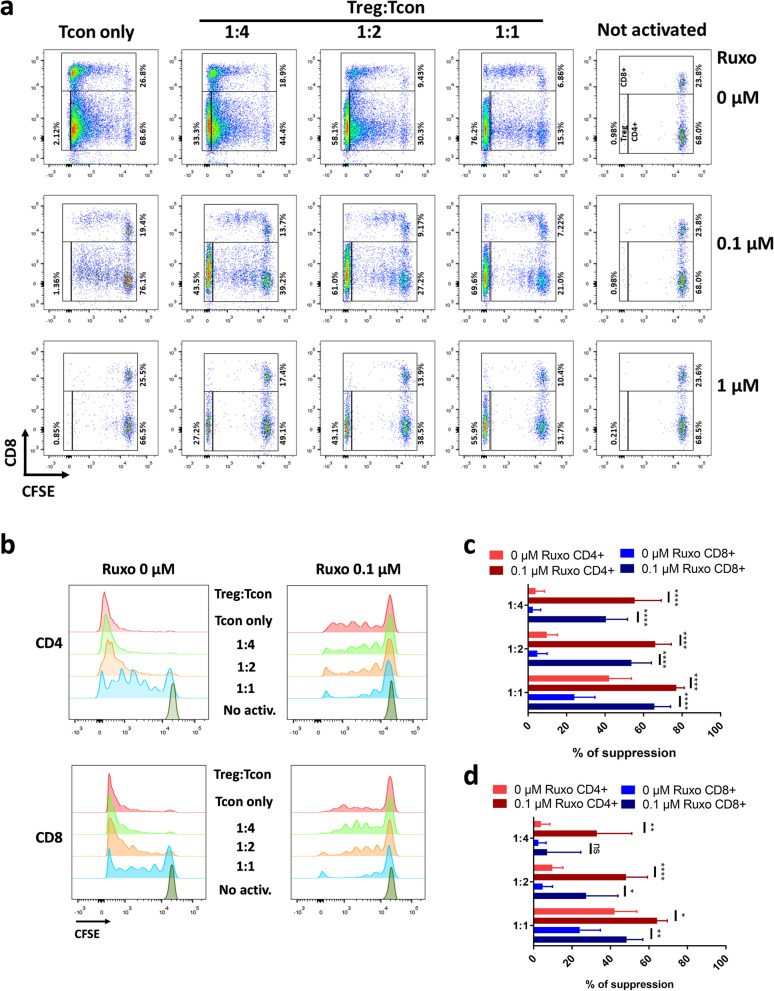
In vitro suppression assays. (**a**) Representative cytometry dot plots showing CD8 and CFSE staining of in vitro suppression assays. huPBMNCs were stained with CFSE, and activated with anti CD3 and anti CD8 stimulation in the presence of different ratios of huTregs and Ruxolitinib. (**b**) Representative histogram plots of the CFSE dilution of CD4+ and CD8+ responder Tcon cells. (**c**) The percentage of suppression of four independent experiments is shown, for CD4+ and CD8+ gated responder cells. The percentage of suppression was calculated using as normalization control the Tcon only ruxolitinib 0 µM samples, to measure the additive effect of Treg and ruxolitinib. Percentage of suppression is calculated as {1 − (%proliferation in the sample/%proliferation in the control)} × 100%. (**d**) As in (**c**), but in this case the values were normalized with the Tcon only Ruxolitinib 0.1 µM for the ruxolitinib treated samples, and the Tcon only Ruxolitinib 0 µM samples for the non-treated samples, to measure only the effect of Tregs. Pairwise p values of a paired Student’s t-test are shown. p values: *< 0.05, **< 0.01, ***< 0.001, ****< 0.0001.

Next, we tested the suppressive capacity of the Treg in the presence of ruxolitinib in an in vitro suppression assay. Magnetic sorted purified human CD4+ CD25+ CD127^low^ Treg were mixed with CFSE labeled huPBMNCs at different ratios, in the presence of different concentrations of ruxolitinib, and with anti-CD3 and anti-CD28 stimulation (Fig. 4). The proliferation of responder T cells was almost completely abolished in the presence of 1 µM of ruxolitinib, independently of the presence or not of Treg (Fig. 4a, lower row). However, at 0.1 µM ruxolitinib, although strongly reduced, cells were still able to proliferate in the absence of Treg, both CD4 and CD8 responders (Fig. 4a,b). The addition of Treg suppressed this proliferation in a dose-dependent manner. The quantification of the suppression capacity normalizing to the non treated controls showed the additive effect of ruxolitinib and Treg (Fig. 4c). More interestingly, when we normalize to the non Treg control of each ruxolitinib treatment, we can observe that the suppression capacity of Treg is not diminished in the presence of 0.1 µM ruxolitinib and, on the contrary, it is even higher (Fig. 4d), suggesting a synergistic effect of ruxolitinib and Treg in the suppression of the activation. We also pretreated purified Treg with ruxolitinib for 24 h, then washed and performed the suppression assay. The results show also that the suppression capacity is enhanced upon treatment of Tregs with ruxolitinib 0.1 µM (Supplementary Fig. 3).

### Progressive onset GvHD mouse model

**Figure 5:**
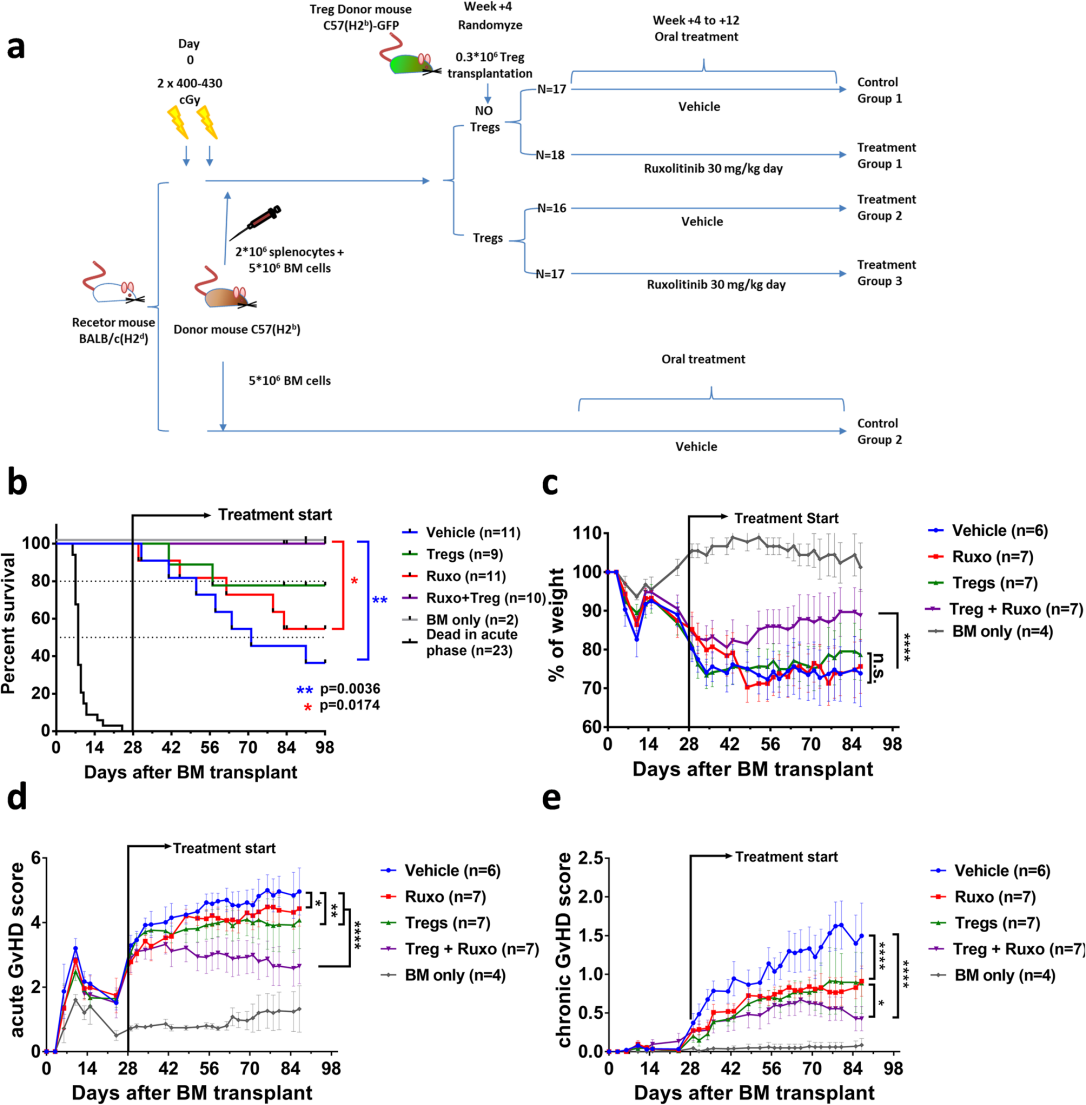
GvHD mouse model. (**a**) Scheme of the mouse model. BALB/c mice were irradiated with 800–860 cGy Split in two doses with 3 h of difference. 4 h later, 2 × 10^6^ splenocytes depleted of monocytes and 5 × 10^6^ BM cells from C57BL/6 donors were transplanted via tail vein injection. Four weeks after transplantation, surviving mice were randomized, and divided in four treatment groups. Treg were purified from GFP mice isogenic to the transplantation donors. 3 × 10^5^ Treg were infused in a single dose to the corresponding groups. The other groups were infused with medium. From this day, animals received ruxolitinib (30 mg/kg-day) or vehicle, via oral gavage. (**b**) Kaplan Meyer representation of the survival of the different treatment groups. A Log-Rank test was used to calculate the p-values of the survival differences between the double treated sample and the other groups. (**c**) Weight loss of the mice with the different treated mice. Statistical differences are calculated using a two way ANOVA test. (**d**) Acute graft versus host disease score of the different treatment groups. (**e**) Chronic graft versus host disease score of the different treatment groups. p values: *< 0.05, **< 0.01, ***< 0.001, ****< 0.0001.

**Figure 6:**
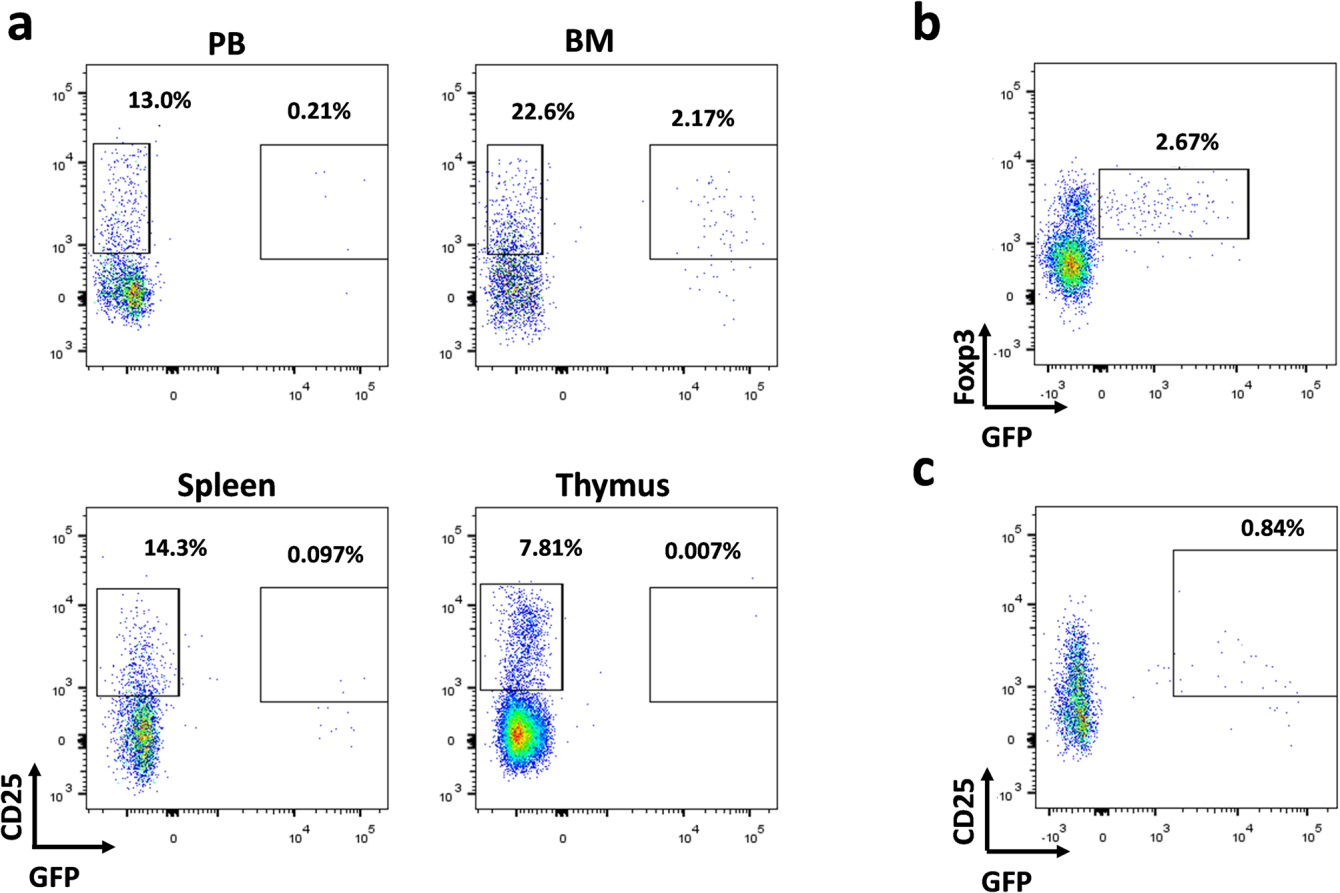
Cytometry analysis of the infused GFP+ Treg in the mice. (**a**) Representative cytometries of cells isolated from different organs (*PB* peripheral blood, *BM* bone marrow, Spleen and Thymus) of a GFP+ Treg infused mouse, sacrificed 10 weeks after infusion. CD3+ CD4+ Gated cells are shown. Infused Treg are detected as CD25+ GFP+ cells. (**b**) Foxp3 staining of the BM cells shown in (**a**). Cells are gated for CD4 expression. GFP+ cells show Foxp3 positive staining. (**c**) Cytometry of bone marrow cells from a GFP+ Treg infused mouse sacrificed 70 weeks post infusion. GFP+ CD25+ cells are still detectable.

To test the therapeutic potential of a combination of ruxolitinib and donor derived Treg in the treatment of GvHD, we decided to perform a preclinical study using a progressive onset GvHD mouse model, in which a first acute GvHD phase is followed by a second phase with signs of both acute and chronic GvHD^56^. We used this model to test the effect of a combined treatment with Treg and ruxolitinib at day 28 post-transplant, once the first acute phase was passed and a second phase of the disease was already stablished. The scheme of treatment is depicted in Fig. 5a. Animals were treated with a single infusion of 3 × 10^5^ GFP Treg isogenic to the donor and/or 30 mg/kg body weight-day of ruxolitinib. The survival (Fig. 5b) of the mice receiving the combined treatment was significantly higher as compared to those receiving the vehicle (p = 0.0036) or the single treatment with ruxolitinib (p = 0.0164), and although not statistically significant, it was also higher than the single treatment with Treg. Weight loss, acute and chronic GvHD clinical scores (Fig. 5c–e) also showed a significantly better behavior in the double treatment group in comparison with the control and the single treatment arms. We took blood samples of the mice at weeks 2, 4 and 6 after the onset of treatment and analyzed by cytometry and hematimetry, and at 12 weeks mice were sacrificed and blood, bone marrow (BM), spleen, thymus, Peyer’s patches, small and large intestine samples were analyzed by flow cytometry (Supplementary Fig. 4, 5). Infused GFP+ Treg (CD4+ CD25+ Foxp3^high^ GFP+) could be detected in the BM and, to a much lower level, in the blood of Treg and Treg + ruxolitinib treated mice (Fig. 6a,b, Supplementary Fig. 4). Histological analysis of the small and large intestine and skin were performed (Supplementary Fig. 6). An improvement of skin pathological scores was observed in mice treated with ruxolitinib and ruxolitinib plus Treg, although it didn’t reach statistical significance. Selected mice were left alive and BM biopsies were performed at weeks 18, 34 and 50 (Supplementary Fig. 4D), and finally sacrificed at week 70 (Fig. 6c). Infused GFP+ Treg survived long-term and were detected in all these time points.

### Graft vs. leukemia effect is not hampered by the combined treatment

**Figure 7:**
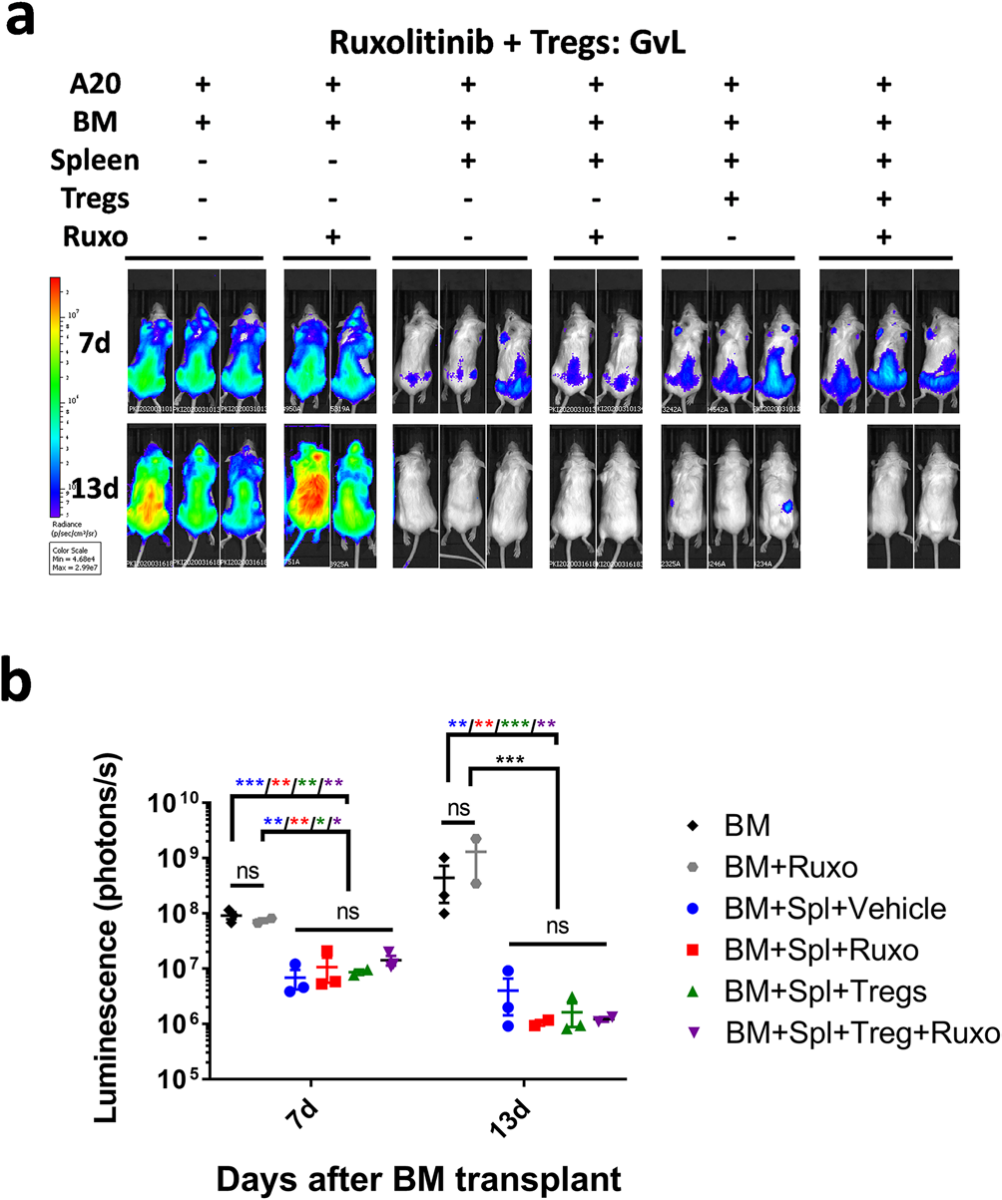
Graft vs. leukemia effect. (**a**) In vivo bioluminescence imaging of BALB/c mice transplanted with BM and Splenocytes from C57BL/6 donors and A20 leukemic cells expressing the Luciferase. Images were obtained at days + 7 and + 13 after transplantation. (**b**) Quantification of the bioluminescence shown in (**a**). Mean and S.E.M is represented in logarithmic scale. Statistical differences are calculated using a two way ANOVA test on the log_10_ bioluminescence values. p values: *< 0.05, **< 0.01, ***< 0.001, ****< 0.0001.

Previous studies have demonstrated that neither ruxolitinib alone or the infusion of Treg interfere with the Graft versus Leukemia effect after transplantation^19,57^. We checked the effect of the combined treatment by infusing Luciferase transduced A20 isogenic leukemic cells into BALB/c recipient mice along with C57 splenocytes (Fig. 7). While in mice not receiving splenocytes the A20 cells proliferated, they did not in mice receiving allogeneic splenocytes in all treatment groups after 2 weeks.

## Discussion

Using in vitro cultures of huPBMNCs, we have shown that upon activation with anti-CD3/CD28, with no treatment, a population of CD25^high^ Foxp3^high^ cells arise in the early stages of the culture, and is strongly reduced along time. Addition of ruxolitinib delays the emergence of this population. Moreover, the presence of ruxolitinib increases the percentage of Helios positive cells. We reason that, in the absence of ruxolitinib, hyperactivation of the culture leads to the apparition of induced Treg from the activated Tcon. On the other hand, the presence of ruxolitinib increases the percentage of Helios+ nTreg in the culture by inhibiting Tcon proliferation without affecting nTreg. This idea is also supported by the inverse correlation of PD1 and Helios expression in these cultures, which could indicate that the Helios− fraction of CD25^high^ FOXP3^high^ originate from exhausted Tcon. It could also be a non suppressive CD25^high^ FOXP3^high^ population, as described previously^58^. Interestingly, in fresh non activated PBMNCs, there is no negative correlation between the expression of Helios and PD1. This result might explain the apparent discrepancies found in previous studies. Spoerl et al.^16^ found that the percentage of Treg is increased in mixed lymphocyte reactions with antigen presenting cells pretreated with low doses of ruxolitinib, while Parampalli Yajnanarayana et al.^53^ found that ruxolitinib impedes the in vitro generation of human iTreg by TGF-β and IL2 polarization. In our case, we have performed the experiments using total PBMNCs activated with anti-CD3 and anti-CD28, but we have obtained equivalent results also with purified CD4+ Tcon.

Ruxolitinib increases the expression of CD39, which is also implicated on immunomodulation via extracellular adenosine production. On the other hand, CTLA4, another important immune checkpoint molecule seems to be downregulated at earlier time points by ruxolitinib, although it is recovered along the culture with higher concentrations of ruxolitinib.

Regarding the expression of chemokine receptor related to gut migration, Ccr9, Ccr5 and Cxcr3, CD4+ Foxp3^high^ cells seem to express higher levels than Foxp3^low^ cells, at all concentrations of ruxolitinib, suggesting that Treg could retain better their homing capacity that Tcon upon treatment, although this effect must be confirmed with in vitro or in vivo migration assays to reach a definitive conclusion.

Stat5, but not Stat3, has been described as an essential factor for the development of Treg^54^. Ruxolitinib inhibits both Stat5 and Stat3 phosphorylation in Tcon^16,53^. However, the effect on Treg suppressive function, once they are generated might be less crucial. In our experimental conditions, ruxolitinib abolished similarly Stat5 and Stat3 phosphorylation in CD4+ Foxp3^high^ cells than in CD4+ Foxp3^low^ and CD8 cells. This was true for both Helios+ and Helios− Foxp3+ cells. This result however does not rule out whether or not in more physiological conditions the phosphorylation of Stat5 could be differentially affected in Treg as compared to Tcon. On the other hand, Stat5 phosphorylation might be important for the generation of Treg, through its role in Foxp3 transcriptional regulation^59^, but it might be dispensable once the Treg are already determined. Conditional knockout or silencing of Stat5 in already differentiated Tregs could help to address these issues.

One important question, not yet addressed, is how ruxolitinib affects the suppressive capacity of Treg. Our in vitro assays indicate that the suppressive capacity is not reduced, and might even be increased, producing more than additive effect. This result together with the fact that ruxolitinib favors the Treg to Tcon ratio both in vitro and in vivo is highly suggestive of a synergistic effect for the treatment of GvHD.

We tested this concept using a GvHD mouse model that reflects the course of the disease in the clinical setting, with an acute phase with high mortality in the early stages followed by a recovery and the subsequent development of a second phase, with characteristics of both acute GvHD, such as weight loss, and chronic GvHD, like skin damage and fibrosis^60^. In contrast to most preclinical studies using ruxolitinib or Treg, we have not started the treatment simultaneously to the BM and splenocytes transplantation but once the early acute phase in our model is passed and the second phase has started to show clinical signs. The fact that GvHD is already in progress instead of using it “prophylactically” might hamper response to treatment, and therefore, in our opinion, makes the results of our study more relevant. In addition to that, we have used reduced doses of both ruxolitinib (30 mg/kg-day compared to the standard dose of 60 mg/kg-day), and 1:6 Treg: splenocyte ratio, instead to the 1:1 or 1:2 ratio used in most studies. We have used these lower doses to detect the possible additive or synergistic effect of both treatment, and to demonstrate that a reduction of both treatments in the combined setting could be beneficial for the patients, reducing side effects of each treatment alone and facilitating to obtain enough Treg. With these settings, we have been able to determine that the combined treatment of GvHD with ruxolitinib and Treg, starting with the disease already established, can outperform the individual treatments, achieving significant higher survival, better clinical scores and lower weight loss, without affecting the Graft versus Leukemia effect. We have also been able to detect the infused GFP+ Treg as long as 70 weeks after infusion, in the bone marrow of Treg treated mice, demonstrating the long-term persistence of the infused Treg. These results have supported the development of a clinical trial, using donor Treg to treat GvHD patients who respond partially to ruxolitinib (NCT03683498).

## Methods

### Human peripheral-blood mononuclear cells (huPBMNCs) purification

Human buffy coats from healthy donors were collected from the Andalusian Health System Biobank with approval of the ethics committee of the University Hospital Virgen del Rocío (1116-n-17). All participants provided informed consent for the use of the samples and all procedures were done in accordance with the Spanish and European regulation and guidelines for research with human samples, and the Declaration of Helsinki. huPBMNCs were purified by ficoll gradient centrifugation.

### Cell culture

huPBMNCs were cultured in RPMI-1640 supplemented with 10% Human AB Serum, Penycilin-Streptomycin and Glutamax. Cells were seeded at a density of 10^6^ cell/ml in 48 well plates. Stimulation was produced with plate bound anti-humanCD3 (BD) (0.5 µg/ml), and soluble anti-humanCD28 (BD) (0.25 µg/ml). Cells were incubated at 37 °C, 5% CO_2_ for the indicated times. Ruxolitinib (INCB018424) was kindly provided by NOVARTIS, and stored in a DMSO stock at 10 mg/ml at – 20 °C.

### Cytometry

Cells were collected, centrifuged and washed in PBS with 2% FCS. For Surface staining, cells were incubated with the corresponding fluorochrome conjugated antibodies (see Supplementary Table 1) for 15 min. at R.T. in the dark. Cells were washed with PBS with 2% FCS, and proceeded to FACs acquisition and analysis, or were processed for intracellular staining. For Intracellular Foxp3 and Helios staining, the Foxp3 staining kit (eBioscience) was used according to the manufacturer’s instructions. For IL10 staining, cells were treated with 10 µg/ml Brefeldin A (Sigma) for 4 h prior to staining. For Phospho-Stat3 and Phospho-Stat5, cells were stained using the BD Phosflow™ T Cell Activation Kit (BD), following manufacturer instructions. Data was acquired in a BD FACS Canto II cytometer, and analyzed using FlowJo v.X software. A minimum of 50,000 events were recorded for each sample. For absolute quantification, 123Count eBeads (Invitrogen) were added to the culture in a 1:50 bead:cell ratio.

### In vitro suppression assays

Human Tregs were isolated from human healthy donors using the CD4+ CD25+ CD127^dim/−^ Regulatory T Cell Isolation Kit II, human (Miltenyi) with an AUTOMACS (Miltenyi) magnetic separator. huPBMNCs responder cells were stained with Carboxyfluorescein succinimidyl ester (CFSE, Invitrogen). 5 × 10^4^ responder were seeded in 96-well plates, and stimulated with anti-humanCD3 and anti-humanCD28. Tregs were added at decreasing ratios, in the presence or absence of ruxolitinib. Proliferation was measured after 5 days by flow cytometry as dilution of CFSE staining. The percentage of suppression was calculated as {1 − (%proliferation in the sample/%proliferation in the control)} × 100%.

### Mice

BALB/c (H-2d) and C57BL/6 (H-2b) mice were purchased from Charles River Laboratories (Morrisville, NC). The green fluorescent protein (GFP) C57BL/6-Tg(ACTB-EGFP)1Osb/J) (H-2b)^61^ were housed in the animal facility of the IBiS. Mice between 7 and 14 weeks old were used. All procedures were approved by the Institutional Animal Care and Use Committee at Institute of Biomedicine in Seville (approval number 09/07/2019/125), and were carried out in compliance with the ARRIVE guidelines.

### Mouse model of GvHD

8–12 week old BALB/c (H-2d) recipient mice were irradiated at day 0 with 800–860 cGy split it two doses separated by 3 h. Mice were transplanted with 5 × 10^6^ BM cells and 2 × 10^6^ splenocytes depleted of monocytes by culturing them for 2 h, from C57BL/6 (H-2b) HLA mismatched donors. After transplantation, first phase of acute GvHD is developed with a moderate percentage of mortality, and after a short recovery period, the surviving mice present a second phase of chronic GvHD^56^. At day 28 post-transplantation, surviving mice were randomized into four treatment groups, receiving a: 3 × 10^5^ Tregs isolated from a GFP transgenic mice isogenic to the donor (C57Bl/6-Tg(ACTB-EGFP)1Osb/J). b: ruxolitinib (30 mg/Kg of body weight once a day) via oral gavage, 5 days a week plus two resting days, c: 3 × 10^5^ Tregs plus ruxolitinib (30 mg/kg once a day) and d: control mice receiving the vehicle of the ruxolitinib and no Treg infusion. Ruxolitinib was prepared at 6 mg/ml in 1:3 PEG 3000:5% Dextrose and administered via oral gavage. Treg were freshly isolated from C57Bl/6-Tg(ACTB-EGFP)1Osb/J) mice spleens using the CD4+ CD25+ Regulatory T Cell Isolation Kit, mouse (Miltenyi). Acute GvHD score was assigned as previously described^62^. Chronic GvHD was adapted from Anderson et al.^63^: Skin damage with fur loss, less than 1 cm^2^ = 1, between 1 and 2.5 cm^2^ = 2, more than 2.5 cm^2^ = 3. Additionally, 0.2 for scaling in the tail, 0.3 points for ear damage and 0.5 points for eye lesions. At week 16 after BM transplantation, animals were sacrificed and exsanguinated. Organs of interest (spleen, liver, skin, Peyer’s patches, small intestine, colon, lung, BM, and thymus) were collected and fixed for histopathological examination. Histopathological scores were assigned by a pathologist according to published scoring system^56^. Cells from peripheral blood, BM, Spleen, Peyer’s patches, large intestine and small intestine were extracted for cytometry analysis as described^56^.

### Mouse model of graft vs. leukemia

8–12 weeks old BALB/c mice were lethally irradiated with 800 cGy split in two doses separated by 3 h. 4 h after irradiation, mice were transplanted with 5 × 10^6^ BM cells, 2 × 10^6^ splenocytes, depleted from monocytes, and 3 × 10^5^ freshly purified Tregs from C57BL/6 donors, and 10^6^ A20 leukemic cells transduced with a GFP-Luciferase vector^64^, depending on the treatment group. Ruxolitinib (30 mg/kg day) or vehicle was administered via oral gavage from day + 1 until the end of the experiment. Luminescence was measured at days + 7 and + 13 using a IVIS Lumina III in vivo imaging system (PerkinElmer, Massachusetts, USA) as previously described^64^.

### Statistics

Data was analyzed using GraphPad PRISM 7.03. Graphs represent Mean and Standard Error of the Mean (S.E.M). Statistical comparisons were made using Student’s t test, one or two way ANOVA test whenever appropriate. Shapiro Wilk test was used to check for normality. Survival curves were represented using the Kaplan Meyer method, and the Log-Rank test was used to determine statistical differences. p values: *< 0.05, **< 0.01, ***< 0.001, ****< 0.0001.

## Supplementary Information

Supplementary Information.

## Data Availability

The datasets used and/or analysed during the current study available from the corresponding author on reasonable request.
