# Supplementary material for: Combined treatment of graft versus host disease using donor regulatory T cells and ruxolitinib
Source: Sci Rep. 2022 May 19;12:8348. doi: 10.1038/s41598-022-12407-x (PMC9120462; doi:10.1038/s41598-022-12407-x)

Supplementary material to

Combined Treatment of Graft versus Host Disease using donor Regulatory T cells and ruxolitinib.

AUTHORS: Alfonso Rodríguez-Gil[1,2,3,4], Virginia Escamilla-Gómez[1,2,3], Melanie Nufer[1], Félix Andújar-Sánchez[1], Teresa Lopes-Ramos[1,2,8], José Antonio Bejarano-García[1], Estefanía García-Guerrero[1,2,3], Cristina Calderón-Cabrera[1,2,3], Teresa Caballero-Velázquez[1,2,3,5], Clara Beatriz García-Calderón[1,3], Paola Hernández-Díaz[1,3], Juan Luis Reguera-Ortega[3], Nancy Rodríguez-Torres[3], Nuria Martínez-Cibrián[3], José Ignacio Rodríguez-Barbosa[6], Javier Villadiego[1,4,7], José Antonio Pérez-Simón[1,2,3,5]

INSTITUTIONS:

[1]Instituto de Biomedicina de Sevilla (IBiS/CSIC/Universidad de Sevilla), Seville, Spain, [2]Centro de Investigación Biomédica en Red en Cáncer-CIBERONC, Madrid, Spain, [3]Hospital Universitario Virgen del Rocío (HUVR), [4]Departamento de Fisiología Médica y Biofísica, Universidad de Sevilla, Seville, Spain, [5]Departamento de Medicina, Universidad de Sevilla, Seville, Spain, [6]Instituto de Biología Molecular, Área de Inmunología, Universidad de León, León, Spain, [7]Centro de Investigación Biomédica en Red sobre Enfermedades Neurodegenerativas-CIBERNED, Madrid, Spain. [8]Present address: Division of Blood and Marrow Transplantation, Stanford University, CA, USA.

Correspondence: Alfonso Rodríguez-Gil, Instituto de Biomedicina de Sevilla (IBiS / CSIC / Universidad de Sevilla) – CIBERONC and Departamento de Fisiología Médica y Biofísica, Universidad de Sevilla. Avda. Manuel Siurot s/n, 41013, Seville, Spain.

E-mail: arg@us.es

Tel: +34 955933087

Fax: +34 955 923101

Supplementary Table 1: Antibodies for Flow Cytometry

Human Antibodies	Provider	Cat. Nr.
**Surface**		
CD4-PerCP Cy5.5	BD	332772
CD8 PE-Cy7	BD	557746
CD8-APC-H7	BD	560179
CD25-FITC	BD	345796
CD25-PE	BD	341011
CD25-CF-BLUE	Immunostep	25CFB-100T
CD127-PE	BD	557938
CD39-BV510	Biolegend	328219
CTLA4-PE-Cy7	Biolegend	349914
CCR5-APC-H7	Biolegend	359110
CCR9-BV421	Biolegend	358913
CXCR3-PE-Cy7	Biolegend	353719
PD1-BV421	Biolegend	329920
**Intracellular**		
FOXP3-APC	Invitrogen	17-4776-42
FOXP3-PE	Invitrogen	12-4776-42
IL10-PE	BD	554706
p-STAT3-Alexa Fluor® 647	BD	557815
p-STAT5-Alexa Fluor® 647	BD	612599
HELIOS-FITC	Biolegend	137214

Mouse Antibodies	Provider	Cat. Nr.
**Surface**		
CD4-APC	BD	553051
CD4-BV421	BD	562891
CD8-PE	Immunostep	MO8APE(V100)
CD19-BV425	Biolegend	115549
CD25-PE-Cy7	BD	552880
CD25-PE	Biolegend	102008
CD45-BV510	BD	533106
CD3-PerCP-Cy5.5	BD	553067
**Intracellular**		
FOXP3-APC	Invitrogen	17-5773-82

Supplementary Figure Legends

Supplementary Figure 1. CD25 and Foxp3 expression, absolute Treg quantification and Tcon:Treg ratio. a. Representative cytometries showing CD25 and Foxp3 staining of CD4+ (blue) and CD8+ (red) cells after anti-CD3/CD28 activation and ruxolitinib treatment. **b.** Absolute quantification of CD4+CD25+Foxp3^{high} cells. Average and S.E.M. of two independent experiments with three technical replicates each are shown. Cells are normalized to counting beads in the culture (1 bead/50 PBMNCs in the initial culture) **c.** Quantification of CD4+ or CD8+ to CD4+CD25+Foxp3^{high} cells ratio . Average and S.E.M. of two independent experiments with three technical replicates each are shown.

Supplementary Figure 2. Correlation of PD1 and Helios, cytometries of CTLA4 and CD39. a. Freshly isolated huPBMNCs were analyzed for Helios and PD1 expression in CD4+ CD25+ Foxp3^{high} Tregs. One representative cytometry is showing the left, and the quantification of 5 independent experiments is shown in the right panel (mean +/-S.E.M.). **b.** Dot plot of the percentage of PD1+ cells versus Helios+ cells of CD4+ Foxp3+ gated cells after 2, 5 and 8 days of antiCD3 and antiCD8 stimulation and Ruxolitinib treatments. The linear regression of each day is shown, with the equation, Pearson correlation (R2) and p value. **c.** Representative cytometry of stimulated huPBMNCs, gated for CD4+, showing CD39 and CTLA4 staining after 2, 5 and 8 days of treatment with 0 or 0.3µM Ruxolitinib.

Supplementary Figure 3. Suppression assay after pretreatment of Treg with Ruxolitinib. MACS sorted Treg were preincubated in the presence of ruxolitinib 0.1 and 0.3 µM for 24h and then ruxolitinib was washed. Later, CD4+ cells labeled with cell division tracking dye were activated with anti CD3 and anti CD28 antibodies and the pretreated Treg were added at different proportions. **a.** CD4+ proliferation was determined by flow cytometry. **b.** The percentage of suppression was calculated as {1-(%proliferation in the sample/%proliferation in the control)}×100%.

Supplementary Figure 4. Cytometry analysis of samples from the GvHD mouse model. Mice were sacrificed 10 weeks after treatment start (14 weeks after BM transplantation) and the cells of different organs were analyzed by cytometry. **a**. Representative cytometries of peripheral blood from BM only transplanted mice (no GvHD), or spleen and BM transplanted cells treated with vehicle, Tregs, Ruxolitinib or both. CD45 gated cells are shown in the upper row for CD3 and CD19 staining, CD3 gated cells are further gated for CD8 and CD4 staining (middle row) and CD4 gated cells are analyzed for CD25 and GFP expression (lower row). **b.** Cells from bone marrow are analyzed as in A. **c.** Cells from BM are stained for Foxp3 expression. Cells are gated for CD4 expression (upper row), and the represented for Foxp3 versus CD25 (middle row) or Foxp3 versus GFP. GFP signal is reduced compared to figure B due to the fixation and permeabilization used for Foxp3 intracellular staining. **d.** Bone marrow biopsies of mice infused with GFP Tregs at the indicated times post-infusion. CD4 gated cells are analyzed for CD25 and GFP expression.

Supplementary figure 5. Quantification of cell populations in the GvHD mouse model. a. Peripheral blood samples were extracted from mice at 2, 4 and 6 weeks after treatment start (6, 8 and 10 weeks post transplantation). Samples of at least 4 mice per group were analyzed following the gating strategy shown in supplemental figure 4A. Mean and S.E.M. is represented. **b.** Mice were sacrificed 10 weeks after treatment start (14 weeks after BM transplantation). Cell populations from peripheral blood, bone marrow (BM), large intestine (LI), Peyer´s patches (PP), small intestine (SI), spleen and thymus were analyzed as in A. **c.** CD25+ Foxp3^{high} Tregs from peripheral blood, bone marrow (BM), spleen and thymus were analyzed following the gating strategy shown in supplementary figure 4C. Mean and S.E.M. is represented. **d.** Blood samples from mice at 2, 4 and 6 weeks after treatment start were analyzed using an haematocytometer. LYM= lymphocytes (10^3/µl), MON=monocytes (10^3/µl), GRA=granulocytes (10^3/µl), WBC= White blood cells (10^3/µl), percent_LYM= percentage of lymphocytes, percent_MON= percentage of monocytes, percent_GRA= percentage of granulocytes. p values of a Student´s t-test are shown. Significant values are marked with red arrows.

Supplementary figure 6. Histopathological analysis of small and large intestine and skin of the GvHD mouse model. Large intestine, small intestine and skin from mice sacrificed after 10 weeks of treatment were fixed in formalin, and included in paraffin. Slices were stained with hematoxylin-eosin and Masson´s trichrome, and were evaluated by a pathologist for GvHD signs. A histopathological score was assigned according to published scoring system[56]. Average plus standard error of the mean of four mice per group are shown. LI, Large Intestine. SI, Small Intestine.

Sup. Fig. 1

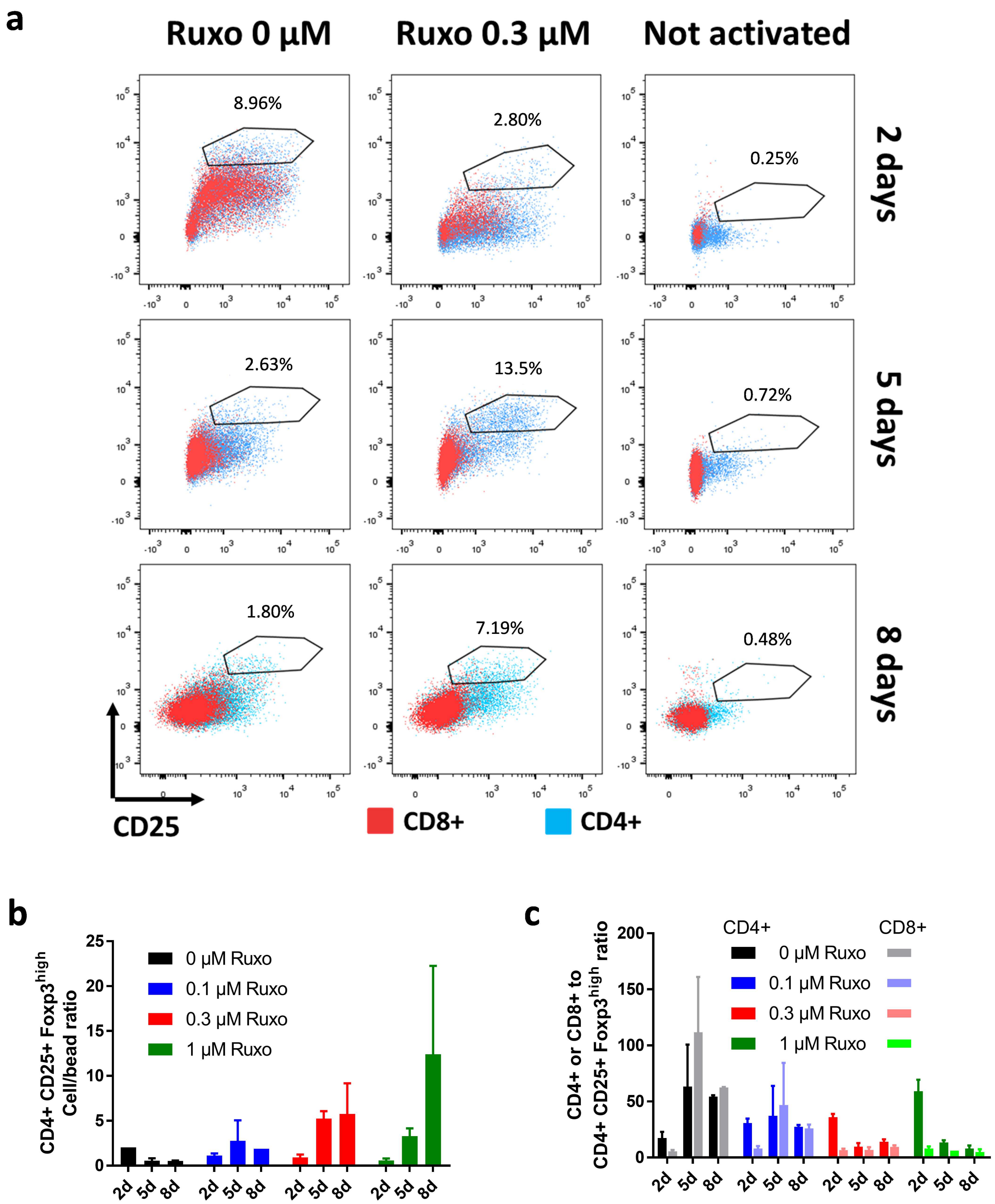
a
Ruxo 0 µM
Ruxo 0.3 µM
Not activated
8.96%
2.80%
0.25%
2 days
2.63%
13.5%
0.72%
5 days
1.80%
7.19%
0.48%
8 days
CD25
CD8+
CD4+
b
CD4+ CD25+ Foxp3high Cell/bead ratio
0 µM Ruxo
0.1 µM Ruxo
0.3 µM Ruxo
1 µM Ruxo
2d
5d
8d
c
CD4+ or CD8+ to CD4+ CD25+ Foxp3high ratio
CD4+
CD8+
0 µM Ruxo
0.1 µM Ruxo
0.3 µM Ruxo
1 µM Ruxo

Sup. Fig. 2

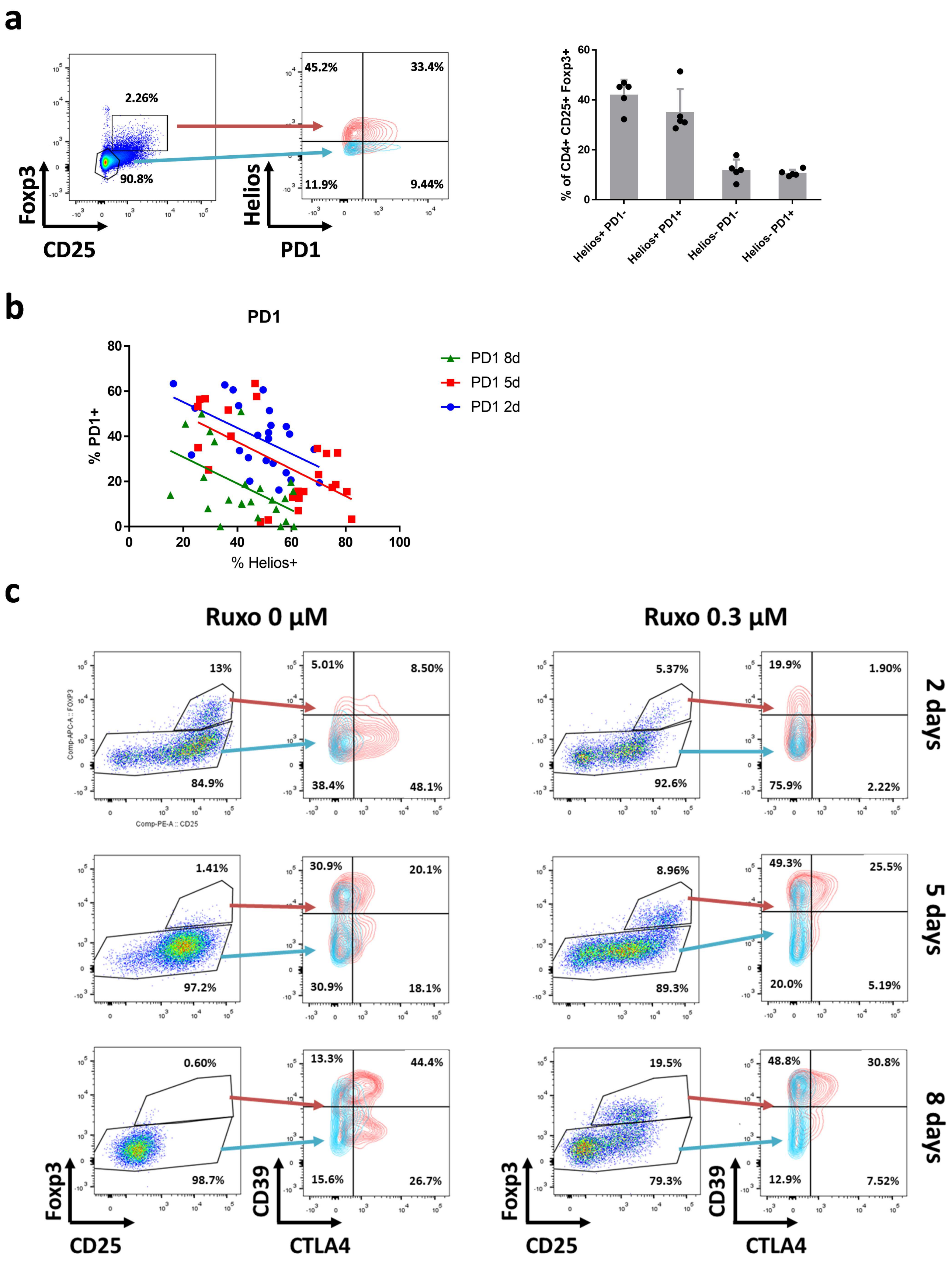
a
Foxp3
CD25
2.26%
90.8%
Helios
PD1
45.2%
33.4%
11.9%
9.44%
% of CD4+ CD25+ Foxp3+
Helios+ PD1-
Helios+ PD1+
Helios- PD1-
Helios- PD1+
b
PD1
% PD1+
% Helios+
PD1 8d
PD1 5d
PD1 2d
c
Ruxo 0 µM
Ruxo 0.3 µM
2 days
5 days
8 days
Comp-APC-A :: FOXP3
Comp-PE-A :: CD25
13%
84.9%
5.01%
8.50%
38.4%
48.1%
5.37%
92.6%
19.9%
1.90%
75.9%
2.22%
1.41%
97.2%
30.9%
20.1%
30.9%
18.1%
8.96%
89.3%
49.3%
25.5%
20.0%
5.19%
0.60%
98.7%
13.3%
44.4%
15.6%
26.7%
19.5%
79.3%
48.8%
30.8%
12.9%
7.52%
CD39
CTLA4

Sup. Fig. 3

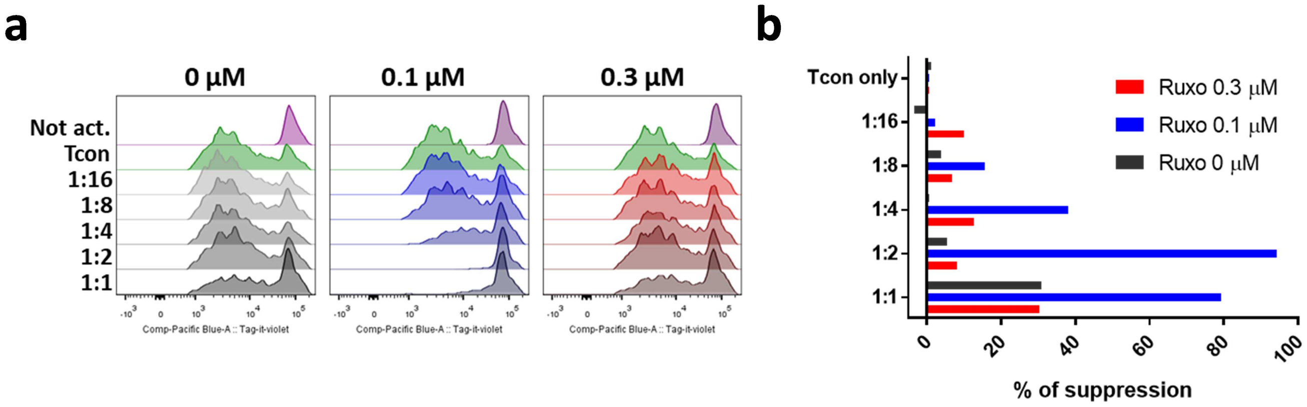
a
0 µM
0.1 µM
0.3 µM
Not act.
Tcon
1:16
1:8
1:4
1:2
1:1
Comp-Pacific Blue-A :: Tag-it-violet
b
Tcon only
1:16
1:8
1:4
1:2
1:1
Ruxo 0.3 µM
Ruxo 0.1 µM
Ruxo 0 µM
0
20
40
60
80
100
% of suppression

Sup. Fig. 4

a. Peripheral blood

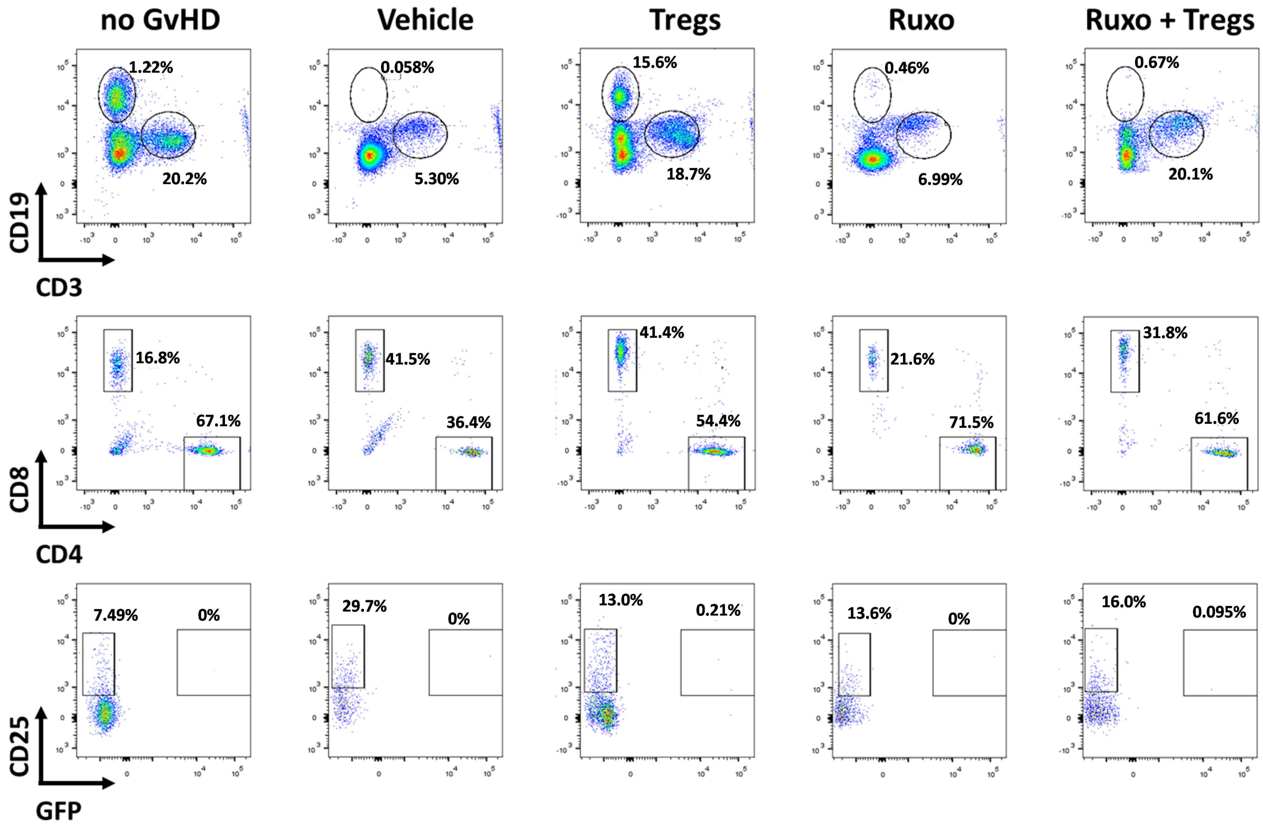

b. Bone Marrow

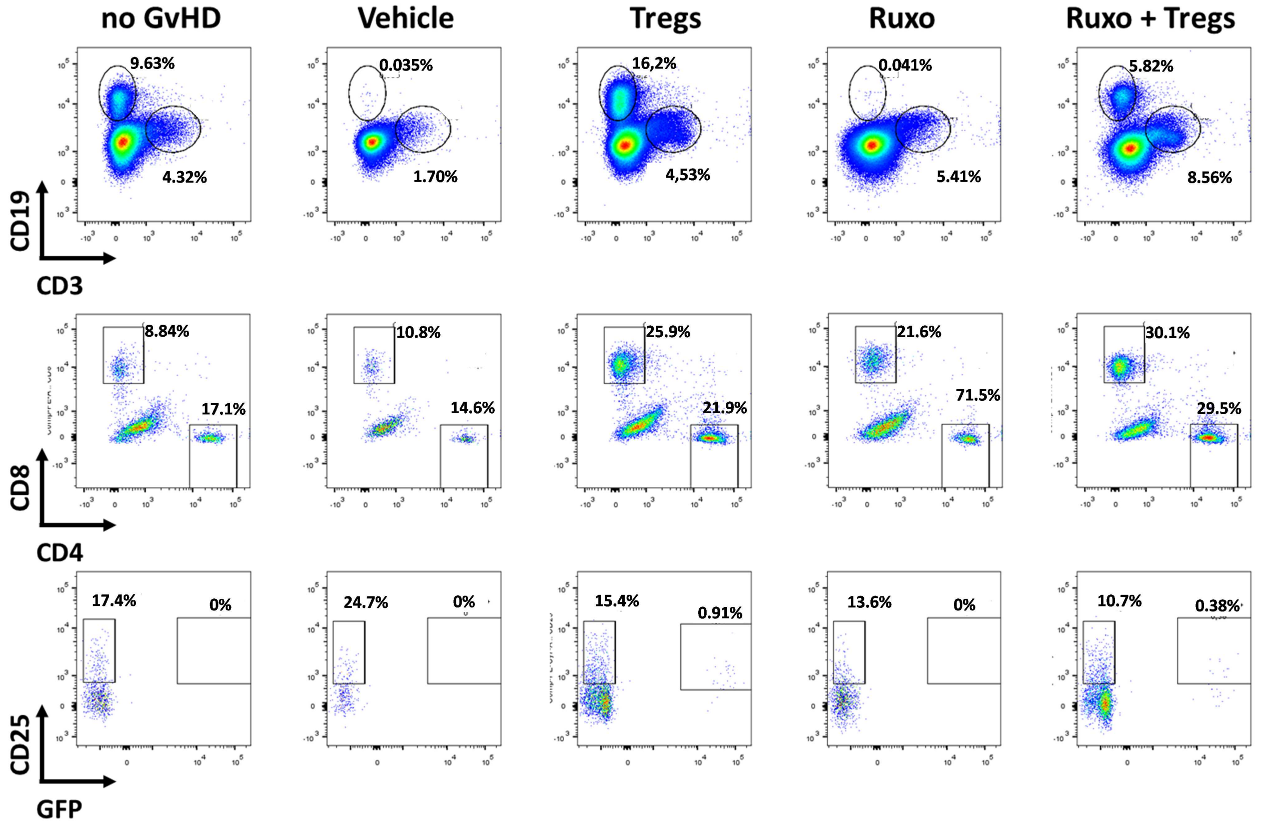

Sup. Fig. 4 (cont.)

c. Bone Marow, Foxp3 Staining

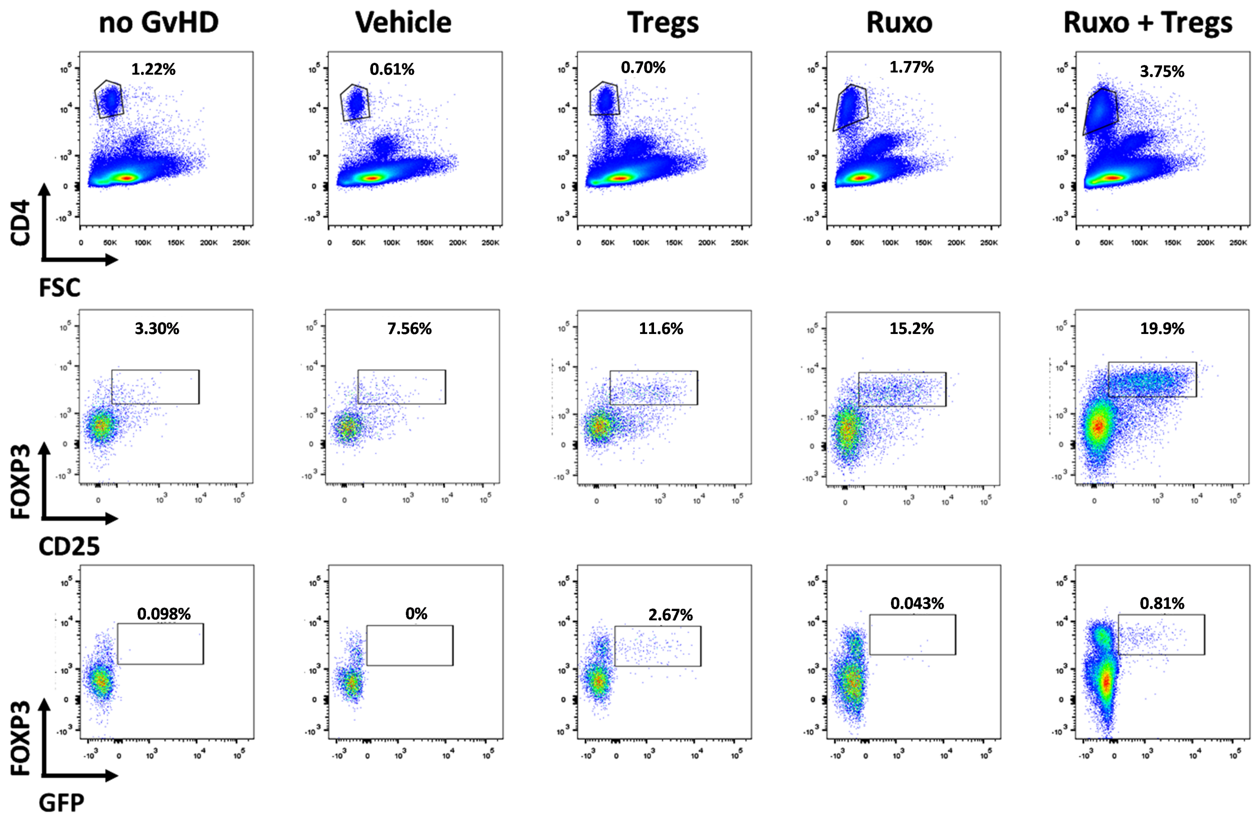

d. Bone Marow biopsies

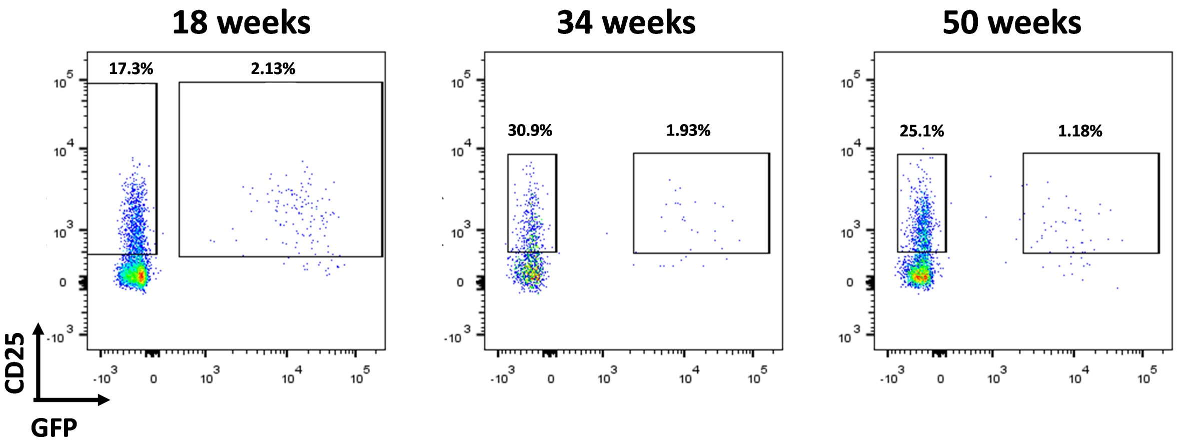

Sup. Fig. 5

a **Ruxolitinib + Tregs: cytometries in PB, weeks 2, 4 and 6 post-treatment**

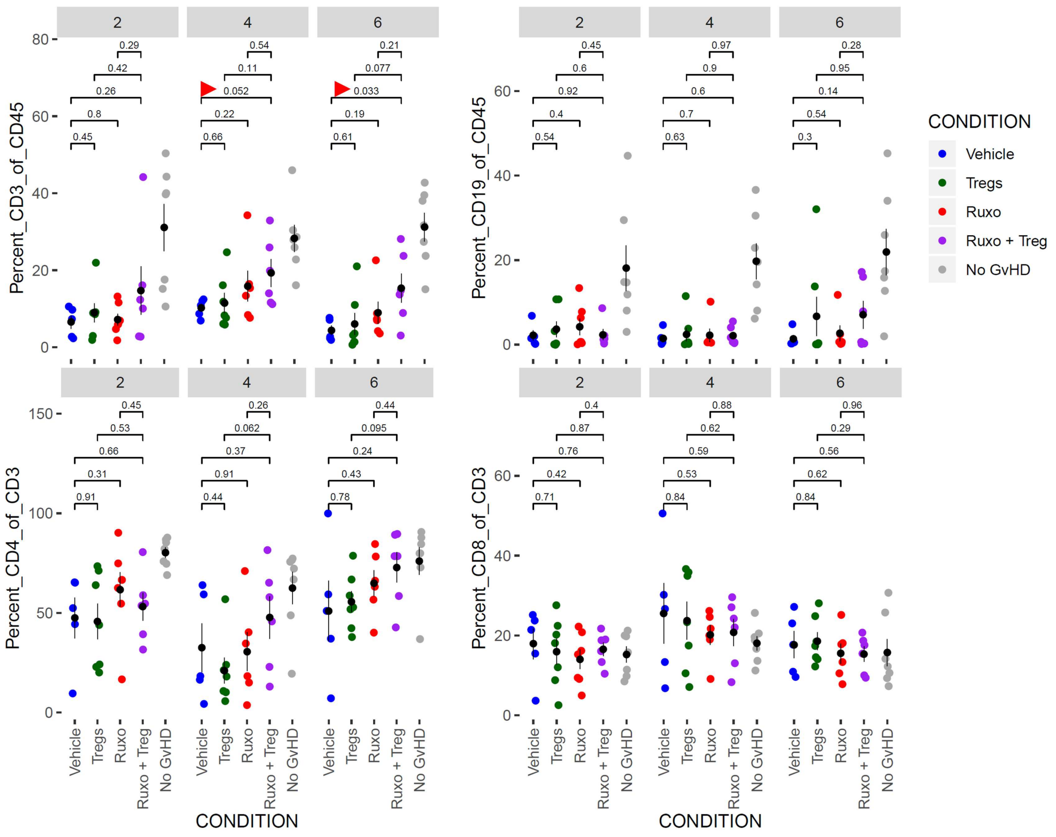

b **Ruxolitinib + Tregs: cytometries at sacrifice (week +10 post treatment)**

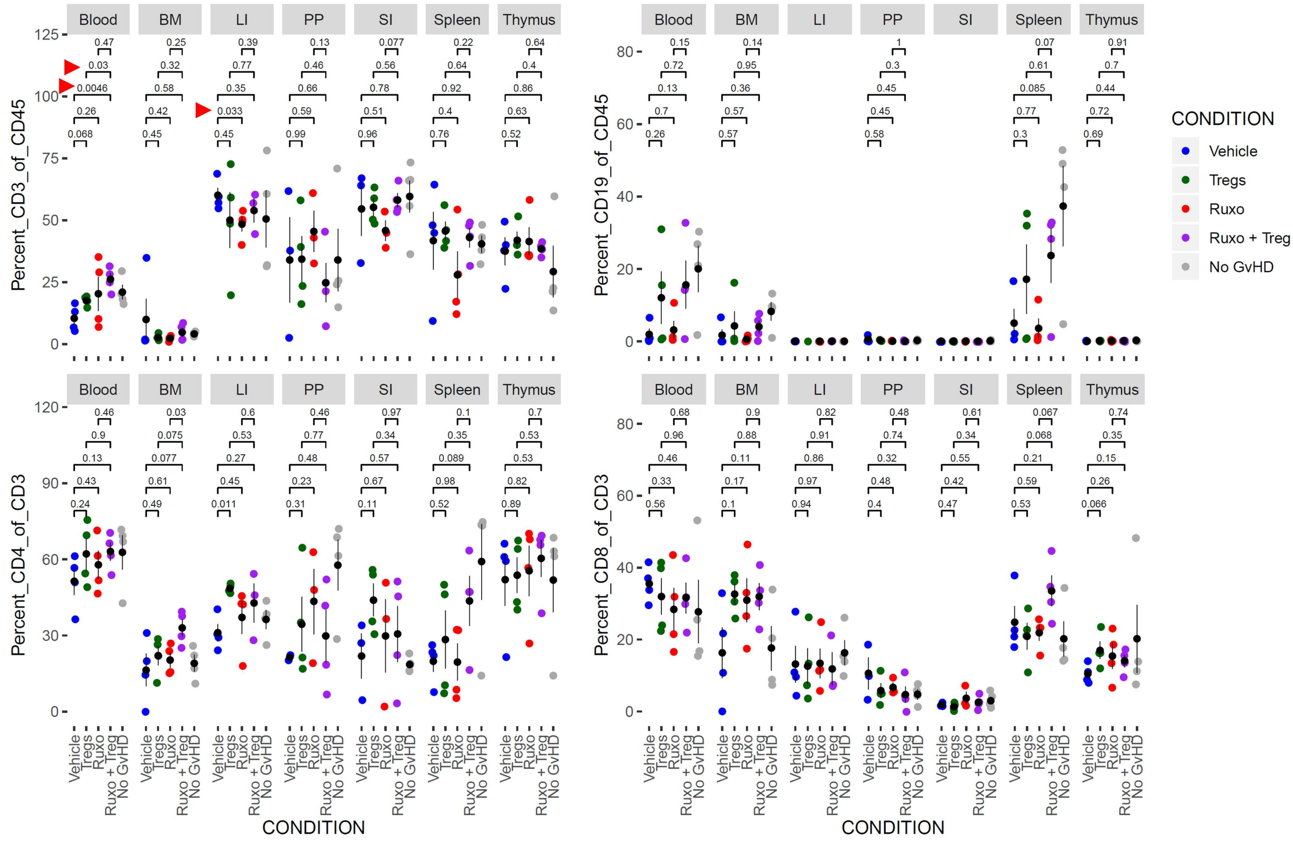

Sup. Fig. 5 (cont.)

c

Ruxolitinib + Tregs: cytometries at sacrifice (week +10 post treatment)

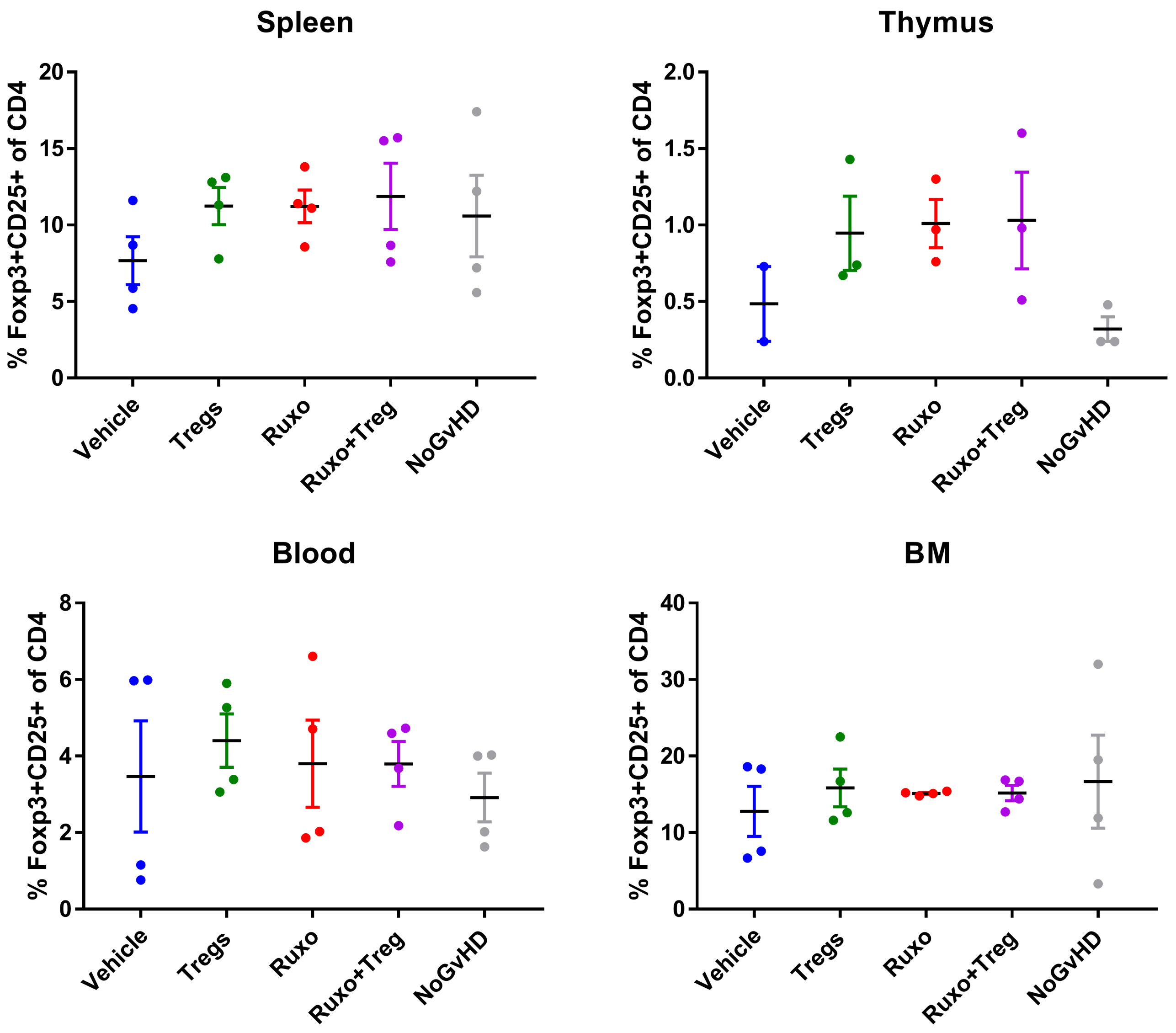

Sup. Fig. 5 (Cont)

d

Ruxolitinib + Tregs: hematimetries

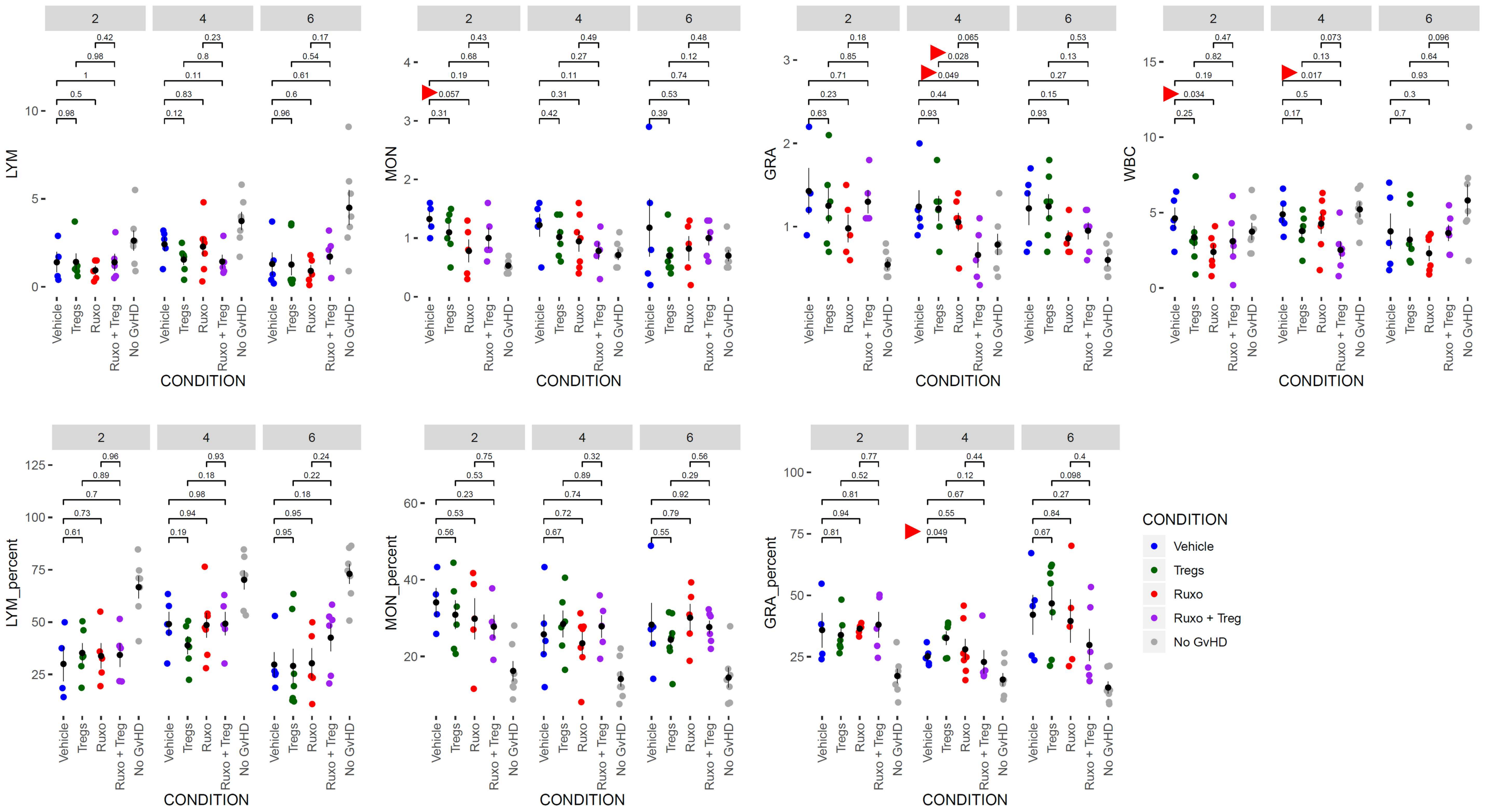

Sup. Fig. 6

c

Ruxolitinib + Tregs: histopathological scores at sacrifice (week +10 post treatment)

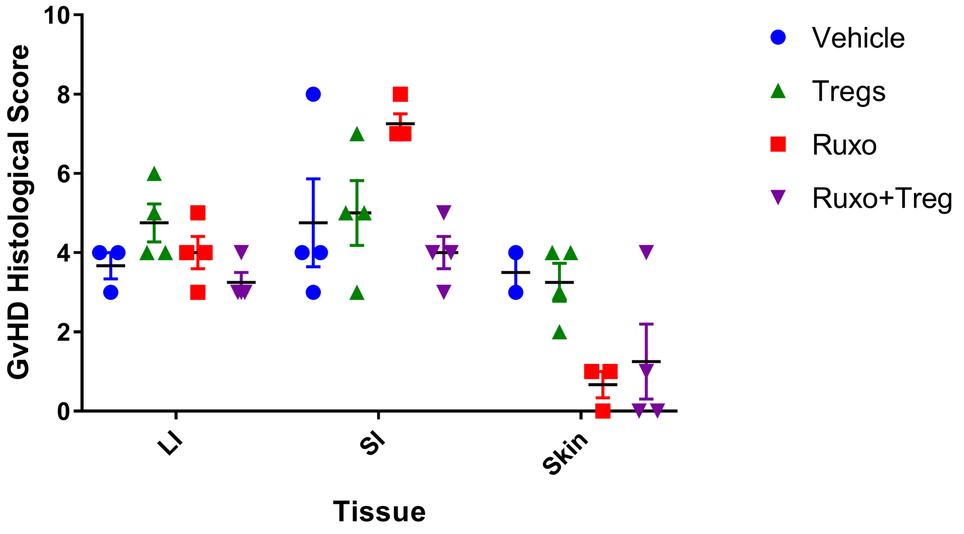

Supplement: Supplementary file 1 — Supplementary Information. [file 41598_2022_12407_MOESM1_ESM.pdf]
